# Supplementary material for: The causal role of circulating inflammatory markers in osteoporosis: a bidirectional Mendelian randomized study
Source: Front Immunol. 2024 Jul 18;15:1412298. doi: 10.3389/fimmu.2024.1412298 (PMC11291241; doi:10.3389/fimmu.2024.1412298)
Supplement: Supplementary file 1 [file DataSheet_1.zip › supplementary material/CIM-OSTEOPOROSIS /GCST90274846.csv_leave_one_out.pdf]

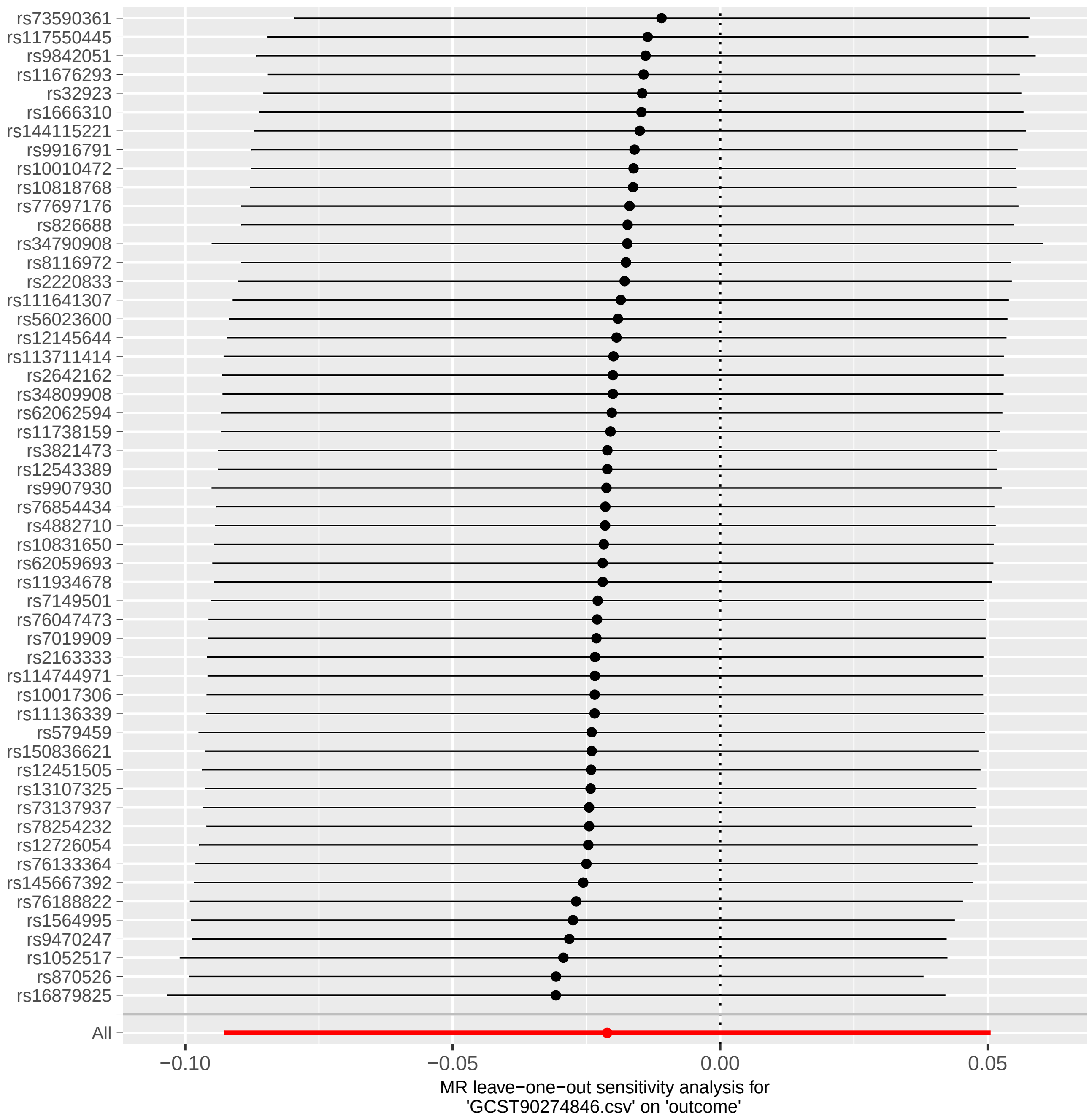
rs73590361
rs117550445
rs9842051
rs11676293
rs32923
rs1666310
rs144115221
rs9916791
rs10010472
rs10818768
rs77697176
rs826688
rs34790908
rs8116972
rs2220833
rs111641307
rs56023600
rs12145644
rs113711414
rs2642162
rs34809908
rs62062594
rs11738159
rs3821473
rs12543389
rs9907930
rs76854434
rs4882710
rs10831650
rs62059693
rs11934678
rs7149501
rs76047473
rs7019909
rs2163333
rs114744971
rs10017306
rs11136339
rs579459
rs150836621
rs12451505
rs13107325
rs73137937
rs78254232
rs12726054
rs76133364
rs145667392
rs76188822
rs1564995
rs9470247
rs1052517
rs870526
rs16879825
All
−0.10
−0.05
0.00
0.05
MR leave−one−out sensitivity analysis for
'GCST90274846.csv' on 'outcome'
